# Association between Chronic Kidney Disease and Sudden Sensorineural Hearing Loss: A Longitudinal Follow-Up Studies Using ICD-10 Codes in a National Health Screening Cohort

**DOI:** 10.3390/jcm12082861

**Published:** 2023-04-14

**Authors:** Ye Ji Shim, Hyo Geun Choi, Jee Hye Wee

**Affiliations:** 1 Department of Otorhinolaryngology-Head and Neck Surgery, Healthcare System Gangnam Center, Seoul National University Hospital, Seoul 06236, Republic of Korea; 2 Sensory Organ Research Institute, Seoul National University Medical Research Center, Seoul 03087, Republic of Korea; 3 SuSeoseoulent Clinic, Seoul 06349, Republic of Korea; 4 Mdanalytics, Seoul 06349, Republic of Korea; 5 Department of Otorhinolaryngology-Head and Neck Surgery, Hallym Sacred Heart Hospital, Hallym University College of Medicine, Anyang 14068, Republic of Korea

**Keywords:** chronic kidney disease, sudden sensorineural hearing loss, cohort study, cardiovascular risk factors, propensity score

## Abstract

This study aims to investigate the association between chronic kidney disease (CKD) and sudden sensorineural hearing loss (SSNHL) using a population-based cohort study. We used data from the Korean National Health Insurance Service–Health Screening Cohort. Participants were selected based on diagnosis and treatment codes, and CKD participants were 1:4 matched with control participants. Covariates, including demographic and lifestyle factors, and comorbidities were considered in the analysis. We calculated the incidence rate and hazards ratio of SSNHL. A total of 16,713 CKD participants and 66,852 matched controls were enrolled. The CKD group had a higher incidence rate of SSNHL compared to the control group at 2.16 and 1.74 per 1000 person-years, respectively. The CKD group exhibited a higher risk for SSNHL compared to the control group with adjusted HR 1.21. In the subgroup analysis, the presence of cardiovascular risk factors was associated with a diminished effect of CKD on the risk of developing SSNHL. This study provides strong evidence of an association between CKD per se and an increased risk of SSNHL even after adjusting for various demographic and comorbidity factors. The findings suggest that CKD patients may require more comprehensive monitoring for hearing loss.

## 1. Introduction

Chronic kidney disease (CKD) is a significant health problem affecting over 10% of the global population, leading to increased morbidity, mortality, and health care costs [1]. CKD is defined as a reduction in kidney function indicated by a glomerular filtration rate (GFR) of less than 60 mL/min per 1.73 m^2^ and/or the presence of markers of kidney damage, such as albuminuria, abnormal urinary sediment, electrolyte, or other abnormalities due to tubular disorders, abnormalities observed in histology examinations, structural abnormalities detected by imaging, or a history of kidney transplantation, of at least 3 months’ duration regardless of underlying cause. CKD is associated with an increased risk of cardiovascular disease, hypertension, and anemia [2]. On the other hand, sudden sensorineural hearing loss (SSNHL) is an abrupt decline of hearing threshold by more than 30 dB in three contiguous frequencies occurring in less than 72 h [3]. The exact etiology of SSNHL remains unclear, but several potential risk factors have been proposed, including viral infections, autoimmune disorders, and vascular events [4].

Interestingly, the cochlea and the kidneys share several physiological mechanisms, such as active transport of fluid and electrolytes and common antigenicity [5]. Consequently, medications and immunological factors may have similar effects on both organs. In renal failure, hearing loss is a common occurrence that has been attributed to various factors, such as electrolyte imbalances, hypertension, hemodialysis treatment, and the use of ototoxic medications [5,6].

Recently, a few reports have suggested a potential link between CKD and SSNHL. Several population-based studies have shown a higher incidence of SSNHL in patients with CKD [7,8,9]. Additionally, some case series have reported clinical presentations of SSNHL in CKD patients [10,11]. Despite these findings, the relationship between CKD and SSNHL remains poorly understood, and the underlying mechanisms have yet to be fully elucidated.

Given the limited research investigating the relationship between CKD and SSNHL, we investigated the association between these two conditions using a population-based cohort study. We utilize data from a national health registry to obtain information on a large and representative sample of the population. Our study aims to shed light on the potential link between CKD and SSNHL and provide further insights into the underlying mechanisms involved.

## 2. Materials and Methods

### 2.1. Ethics

The ethics committee of Hallym University (2019-10-023) permitted this study. Written informed consent was waived by the Institutional Review Board. All analyses adhered to the guidelines and regulations of the ethics committee of Hallym University. 

### 2.2. Data Collection

The data from the Korean National Health Insurance Service–Health Screening Cohort was used. The detailed description of the Korean National Health Insurance Service–Health Screening Cohort data is described in [12]. Briefly, the KNHIS-HSC dataset comprises health insurance claim codes for procedures and prescriptions, diagnostic codes utilizing the International Classification of Disease-10 (ICD-10), death records, socioeconomic data, and health checkup data (including body mass index [BMI], drinking and smoking habits, blood pressure, urinalysis, hemoglobin, fasting glucose, lipid parameters, creatinine, and liver enzymes).

### 2.3. Exposure (Chronic Kidney Disease)

CKD was categorized if the participants were diagnosed with CKD (ICD-10 codes: N18) ≥ 2 times or unspecified kidney failure (ICD-10 codes: N19). Also, participants were included if they had received regular dialysis treatment (hemodialysis and/or peritoneal dialysis), treatment codes (O7010, O7020 and O7070). In this study, we did not differentiate between the different stages of CKD due to the limitations of available data.

### 2.4. Outcome (Sudden Sensorineural Hearing Loss)

SSNHL was defined if the participants were visited with due to diagnosis of ICD-10 codes H912 (SSNHL). We only included the participants who underwent audiometric examination (claim code: E6931-E6937, F6341-F6348) and used steroid treatment.

### 2.5. Participant Selection

**Figure 1:**
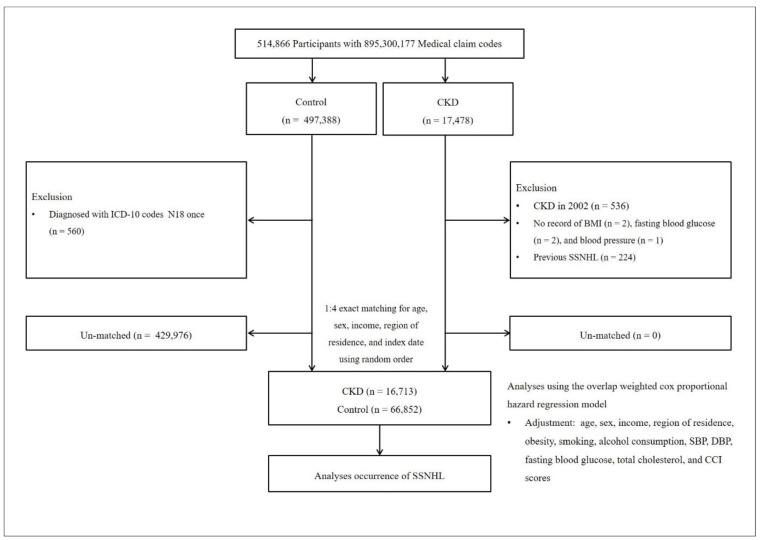
A schematic illustration of the participant selection process that was used in the present study. Of a total of 514,866 participants, 16,713 of CKD participants were matched with 66,852 of control participants for age, sex, income, and region of residence.

CKD participants were selected from 514,866 participants with 895,300,177 medical claim codes from 2002 through 2019 (*n* = 17,478). The control group was included if participants were not defined as CKD from 2002 through 2019 (*n* = 497,388). To select the CKD participants who were diagnosed the first time, CKD participants diagnosed in 2002 were excluded (washout periods, *n* = 536). CKD participants who have no record of BMI (*n* = 2), fasting blood glucose (*n* = 2), and blood pressure (*n* = 1) were excluded. Control participants who were diagnosed with ICD-10 codes N18 once were excluded (*n* = 560). CKD participants were 1:4 matched with control participants for age, sex, income, and region of residence. To prevent selection bias when choosing the matched participants, the control participants were sorted using a random number order and were then selected from top to bottom. It was assumed that the matched control participants were being evaluated at the same time as each matched CKD participant (index date). Therefore, the participants in the control group who died before the index date were excluded. In both the CKD and control groups, the participants who had a history of SSNHL before the index date were excluded. In the CKD group, 224 participants were excluded (left-truncated). During the matching procedure, 429,976 of control participants were excluded. Finally, 16,713 of CKD participants were 1:4 matched with 66,852 control participants (Figure 1). 

### 2.6. Covariates

Age groups were divided into 5-year intervals: 40–44…, and 85+ years old. A total of 10 age groups were specified. Income groups were classified as 5 classes (Class 1 [lowest income]–5 [highest income]). The region of residence was grouped into urban and rural areas following our previous study [13]. Tobacco smoking, alcohol consumption, and obesity using BMI (body mass index, kg/m^2^) were categorized in the same way as our study [14]. The records of systolic blood pressure (SBP, mmHg), diastolic blood pressure (DBP, mmHg), fasting blood glucose (mg/dL), and total cholesterol (mg/dL) were used.

The Charlson Comorbidity Index (CCI) has been used widely to measure disease burden based on 17 comorbidities. A score was given to each participant depending on the severity and number of diseases. The CCI was measured as the continuous variable (0 [no comorbidities] through 29 [multiple comorbidities]) [15,16]. In our study, we excluded CKD (ICD-10 codes: N18 and N19) from the CCI score. 

### 2.7. Statistical Analyses

We carried out the propensity score (PS) overlap weighting to reflect the covariate balance and effective sample size. PS was calculated by multivariable logistic regression with all covariates. To calculate overlap weighting, PS was applied in which CKD participants were weighted by the probability of PS, and control participants were weighted by the probability of 1-PS. Overlap weightings, calculated between 0 and 1, achieve exact balance and optimize precision [17,18,19]. The standardized difference after weighting and before weighting were used to compare the difference of general characteristics between CKD and control groups.

To analyze the overlap weighted hazard ratios (HRs) of CKD for SSNHL, the PS overlap weighted cox proportional hazard regression model was used. In these analyses, crude (unadjusted) and overlap weighted model (adjusted for age, sex, income, region of residence, obesity, smoking, alcohol consumption, SBP, DBP, fasting blood glucose, total cholesterol, and CCI scores) were used.

**Figure 2:**
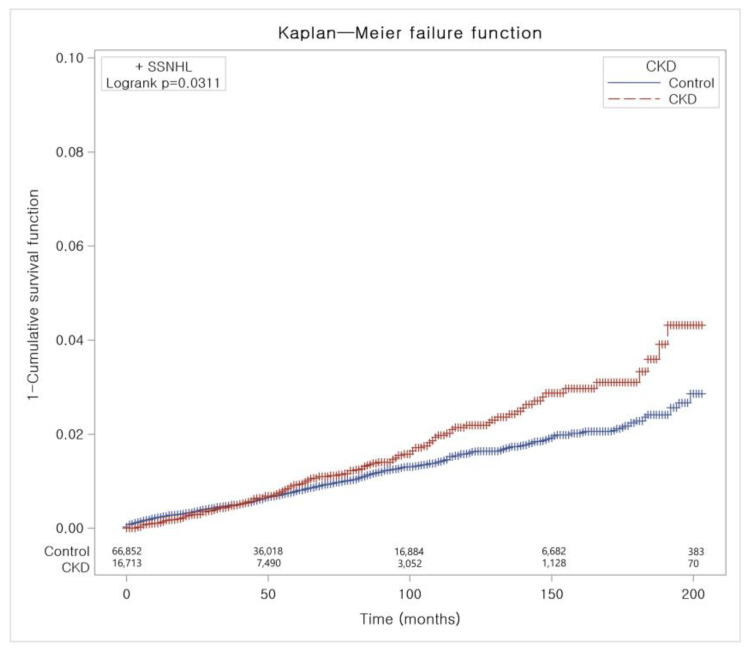
Risk of development of SSNHL among individuals with and without CKD. Abbreviation: SSNHL, sudden sensorineural hearing loss; CKD, chronic kidney disease.

The crude incidence rates (IR) and incidence rates difference (IRD) were calculated by dividing the number of participants with a given event by person-years, which were expressed as cases per 1000 person-years. Kaplan–Meier analysis was conducted to compare the incidence of SSNHL between the CKD group and the control group with log-rank tests (Figure 2). Two-tailed analyses were performed, and significance was defined as *p* values less than 0.05. The SAS version 9.4 (SAS Institute Inc., Cary, NC, USA) was used for statistical analyses.

## 3. Results

A total of 16,713 CKD participants and 66,852 matched controls were enrolled in the current study. The general characteristics of the study participants are presented in Table 1. There was a higher proportion of males, and the majority of participants were in the nonsmoker bracket in both groups. Prior to overlap weighting adjustment, there were no significant differences in age, sex, income, and region of residence between the CKD and control groups. Covariates except for fasting blood glucose and CCI score were equivalent between the two groups (standardized difference ≤ 0.2). The standardized differences for fasting blood glucose and the CCI score are 0.29 and 0.53, respectively, indicating a significant difference. However, after overlap weighting adjustment, the standardized differences for all variables are reduced to 0.00, indicating that there are no significant differences between the two groups after adjustment. SSNHL occurred in 153 (0.92%) and 603 (0.90%) in the CKD group and the control group, respectively. 

The CKD group exhibited a higher IR of SSNHL at 2.16 per 1000 person-years compared to the control group, at 1.74 per 1000 person-years (IRD: HR 0.42, 95% CI: 0.07–0.76) (Table 2). After adjusting all relevant demographics and comorbidities, the CKD group displayed a significantly higher risk for SSNHL compared to the control group (HR 1.21, 95% CI 1.04–1.39; *p* = 0.011). Kaplan–Meier analysis with log-rank test demonstrated a significantly higher cumulative incidence of SSNHL in the CKD group over a 17-year period when compared to the control group (Figure 2). 

The results of the subgroup analysis showed that the risk of SSNHL in CKD participants varied according to different subgroups (Table 2). The CKD group had a significantly higher HR for SSNHL in several subgroups after overlap weighting adjustment (*p* < 0.05). These subgroups included individuals who were under 70 years old, had a high income, lived in urban areas, had a normal or overweight BMI, were non-smokers, consumed alcohol less than once a week, had SBP below 140 mmHg and DBP below 90 mmHg, had fasting blood glucose levels of 100 mg/dL or higher, had total cholesterol levels below 200 mg/dL, and had a CCI score of 0. There were no significant differences in the other subgroups.

## 4. Discussion

The results of our study demonstrate a significant association between CKD and SSNHL. Our findings indicate that individuals with CKD have a higher risk of developing SSNHL compared to controls even after adjusting for various demographic and comorbidity factors.

This finding is consistent with previous population-based cohort studies that have also reported an increased risk of SSNHL in CKD patients [7,8,9]. There are two studies utilizing the National Health Insurance Research Database of Taiwan. Wang et al. compared a cohort of ESRD patients receiving dialysis to a control group and found that the incidence of SSNHL was higher in the ESRD cohort than in the control group, with a HR of 2.71 [9]. Lin et al. reported that the incidence of SSNHL was 1.57 times higher in the CKD group compared to the control group, with an adjusted HR of 1.46 [8]. The study conducted in Korea using the National Health Insurance Service–National Sample Cohort demonstrated that individuals with CKD had a 3.5 times higher incidence of SSNHL compared to those without CKD, and the adjusted HR for developing SSNHL in the CKD group was 2.15, suggesting that CKD is a significant risk factor for SSNHL [7].

Our study employed several methodological improvements over previous research. First, we used PS overlap weighting to correct for potential biases in the analysis, which enabled us to adjust for any potential confounding variables and provide a more accurate representation of the true relationship between CKD and SSNHL. Compared to previous studies that only included patients diagnosed with CKD during a limited time period, our study included all patients with CKD diagnosed from 2002 to 2019. This allowed us to capture a larger sample size and a longer follow-up period, providing a more comprehensive understanding of the association between CKD and SSNHL. Moreover, unlike other studies that relied solely on ICD codes, we specifically focused on patients diagnosed with SSNHL who received audiometric testing and steroid treatment, which allowed for more precise diagnosis and improved the reliability of the results. In contrast to other studies, which did not thoroughly address cardiovascular risk factors that could potentially contribute to the link between CKD and SSNH, our study incorporated a combination of comorbidities, lifestyle data, and laboratory measurements, such as smoking, alcohol consumption, BMI, blood pressure, fasting blood glucose, and total cholesterol levels. By adjusting for potential confounding factors that could impact the relationship between CKD and SSNHL, our approach enabled us to more accurately evaluate the independent effect of CKD on the risk of developing SSNHL.

The pathogenic mechanisms connecting CKD and SSNHL are not fully understood, but several potential explanations have been suggested. One potential explanation is that the increased risk of SSNHL in CKD patients is related to the vascular complications associated with CKD. This is supported by the observation that patients with CKD have an increased risk of cardiovascular complications, likely due to the presence of unique conditions such as anemia, hyperparathyroidism, hyperhomocysteinemia, subclinical systemic inflammation, and increased oxidative stress, in addition to conventional risk factors for cardiovascular diseases [20]. As CKD progresses, it can lead to endothelial dysfunction, which is characterized by impaired endothelium-dependent vasodilation, increased endothelial permeability, and a proinflammatory state. This endothelial dysfunction can lead to reduced blood flow to various organs, including the cochlea. Elevated levels of intercellular and vascular adhesion molecules (ICAM1, VCAM1), which are considered markers of endothelial dysfunction, have been observed in patients with CKD [21,22,23,24,25]. There are studies indicating that levels of circulating adhesion molecules, specifically ICAM-1 and VCAM-1, are elevated in patients with SSNHL [26,27]. This finding suggests the presence of endothelial dysfunction in patients with SSNHL and implies the vascular involvement in the pathogenesis of the disease. Another consequence of endothelial dysfunction is impaired vasodilation. In chronic renal failure, elevated levels of asymmetrical dimethylarginine (ADMA), a competitive inhibitor of eNOS, can lead to a reduction in NO production and hinder endothelial vasodilation in the kidney [28]. Notably, a study has revealed that ADMA is associated with hearing level in CKD patients, suggesting a possible association between endothelial dysfunction and hearing impairment related to CKD [29].

As CKD advances, arterial stiffness and atherosclerosis can also develop, further contributing to impaired blood supply to the cochlea. Arterial stiffness and atherosclerosis in CKD are caused by a combination of uremia-associated risk factors, including the deposition of extracellular matrix proteins, chronic volume overload, inflammation, oxidative stress, and several other factors [30,31].

Another possible explanation is that SSNHL may result from uremic neuropathy, which can develop in patients with CKD due to the accumulation of uremic toxins and electrolyte imbalances. Uremic neuropathy can affect various parts of the nervous system, including the auditory nerve. Damage to the auditory nerve’s axons caused by uremic neuropathy can lead to hearing loss [32,33]. In an animal study, delayed latency of the first wave and similar interpeak I-V latencies for the control were observed in uremic rats, suggesting problems with neural conduction along the acoustic nerve [34]. Studies comparing auditory brainstem response (ABR) between CKD patients and healthy controls have also shown that ABR latency is prolonged in CKD patients, indicating delayed neural conduction along the auditory pathway [35,36,37].

Our subgroup analysis revealed that the increased risk of SSNHL associated with CKD was not observed consistently across all subgroups. In particular, no significant association was found among individuals aged 70 or older defined as low income, rural residence, underweight, obese, history of smoking, alcohol consumption more than once a week, hypertension, dyslipidemia, and a CCI score greater than one.

In the subgroup analysis, it is important to consider the potential influence of cardiovascular risk factors in CKD patients (such as hypertension, dyslipidemia, and obesity) on the observed associations with SSNHL. CKD patients with cardiovascular risk factors may already have a higher baseline risk of developing SSNHL, which could diminish the additional impact of CKD on this risk. In contrast, CKD patients without cardiovascular risk factors may have a lower baseline risk of SSNHL, making the effect of CKD more noticeable. This is consistent with our finding that CKD patients without cardiovascular risk factors had a higher HR for SSNHL than those with risk factors. One of the mechanisms for idiopathic SSNHL is vascular insult. The blood supply to the inner ear depends solely on the labyrinthine artery without collateral flow, making it vulnerable to ischemic events [38]. Some studies have shown an increased incidence of cerebral ischemia in SSNHL patients [39,40]. Cardiovascular diseases such as stroke and ischemic heart disease are caused by atherosclerosis, which may be triggered by traditional cardiovascular risk factors such as hypertension, dyslipidemia, and DM [41]. Previous studies have revealed an increased risk of SSNHL in the presence of cardiovascular risk factors [38,42,43,44,45]. Therefore, when these factors are already present, the impact of CKD on the risk of SSNHL may not be significant. However, our result showed that CKD had an impact on SSNHL when fasting blood glucose was over 100 mg/dL, which is likely due to the fact that diabetes is a common cause of CKD [9].

Our study has some limitations that should be considered. First, our participant selection was based on diagnostic codes and only included individuals from Korea. Second, the lack of comparison for several risk factors, such as the use of ototoxic medications, could have introduced bias into our study. Third, our study did not distinguish between different stages of CKD or different degrees of SSNHL. Future studies could investigate the relationship between different stages of CKD and the severity of SSNHL as well as the risk of developing CKD or SSNHL.

Despite this, our study has several strengths. First, this study used a large sample size from a national cohort in Korea, which enhances the generalizability of the findings. The KNHIS-HSC dataset has been commonly used in population-based studies, since it represents a nationwide population database that includes information from all clinics and hospitals in South Korea [46,47]. Second, we employed strict selection criteria for both the CKD and control groups and conducted thorough matching to reduce potential selection bias. Third, we adjusted multiple confounding variables including demographic factors, lifestyle factors, comorbidities, and laboratory measurements, which strengthens the validity of the results. Fourth, this study used PS overlap weighting, which can improve covariate balance and increase the effective sample size, resulting in more precise estimates of the association between CKD and SSNHL.

## 5. Conclusions

This study provides evidence of an association between CKD and an increased risk of SSNHL. Future studies are needed to confirm whether there is a causal relationship between CKD and SSNHL and to investigate the impact of CKD management on the incidence of SSNHL. Furthermore, future studies should consider potential unmeasured confounders such as noise exposure and medication use that may affect the association between CKD and SSNHL. Distinguishing between different stages of CKD as well as different subtypes of SSNHL may also provide additional insights into the association. Finally, investigating the severity, duration, and response to treatment of SSNHL in patients with CKD may have important clinical implications.

## Figures and Tables

**Table 1:** General Characteristics of Participants.

Characteristics	Before Overlap Weighting Adjustment			After Overlap Weighting Adjustment		
	CKD	Control	Standardized Difference	CKD	Control	Standardized Difference
Age (*n*, %)			0.00			0.00
40–44	98 (0.59)	392 (0.59)		75 (0.60)	75 (0.60)	
45–49	359 (2.15)	1436 (2.15)		260 (2.10)	260 (2.10)	
50–54	943 (5.64)	3772 (5.64)		685 (5.53)	685 (5.53)	
55–59	1846 (11.05)	7384 (11.05)		1353 (10.91)	1353 (10.91)	
60–64	2272 (13.59)	9088 (13.59)		1659 (13.38)	1659 (13.38)	
65–69	2565 (15.35)	10,260 (15.35)		1886 (15.21)	1886 (15.21)	
70–74	2939 (17.59)	11,756 (17.59)		2190 (17.66)	2190 (17.66)	
75–79	2861 (17.12)	11,444 (17.12)		2149 (17.32)	2149 (17.33)	
80–84	1903 (11.39)	7612 (11.39)		1434 (11.57)	1434 (11.57)	
85+	927 (5.55)	3708 (5.55)		711 (5.73)	711 (5.73)	
Sex (*n*, %)			0.00			
Male	10,981 (65.70)	43,924 (65.70)		8158 (65.78)	8158 (65.78)	
Female	5732 (34.30)	22,928 (34.30)		4244 (34.22)	4244 (34.22)	
Income (*n*, %)			0.00			0.00
1 (lowest)	2928 (17.52)	11,712 (17.52)		2162 (17.43)	2162 (17.43)	
2	1924 (11.51)	7696 (11.51)		1429 (11.52)	1429 (11.52)	
3	2384 (14.26)	9536 (14.26)		1763 (14.22)	1763 (14.22)	
4	3308 (19.79)	13,232 (19.79)		2445 (19.72)	2445 (19.72)	
5 (highest)	6169 (36.91)	24,676 (36.91)		4603 (37.11)	4603 (37.11)	
Region of residence (*n*, %)			0.00			0.00
Urban	7157 (42.82)	28,628 (42.82)		5310 (42.82)	5310 (42.82)	
Rural	9556 (57.18)	38,224 (57.18)		7092 (57.18)	7092 (57.18)	
Obesity † (*n*, %)			0.15			0.00
Underweight	443 (2.65)	2220 (3.32)		347 (2.79)	347 (2.79)	
Normal	5129 (30.69)	23,822 (35.63)		3926 (31.66)	3926 (31.66)	
Overweight	4379 (26.20)	18,037 (26.98)		3280 (26.45)	3280 (26.45)	
Obese I	6000 (35.90)	20,871 (31.22)		4345 (35.04)	4345 (35.04)	
Obese II	762 (4.56)	1902 (2.85)		503 (4.06)	503 (4.06)	
Smoking status (*n*, %)			0.02			0.00
Nonsmoker	10,688 (63.95)	43,275 (64.73)		7960 (64.18)	7960 (64.18)	
Past smoker	1748 (10.46)	6965 (10.42)		1304 (10.51)	1304 (10.51)	
Current smoker	4277 (25.59)	16,612 (24.85)		3138 (25.31)	3138 (25.31)	
Alcohol consumption (*n*, %)			0.07			0.00
<1 time a week	12,152 (72.71)	46,344 (69.32)		8910 (71.85)	8911 (71.85)	
≥1 time a week	4561 (27.29)	20,508 (30.68)		3491 (28.15)	3491 (28.15)	
SBP (Mean, SD)	131.85 (18.38)	128.66 (16.25)	0.18	130.87 (15.41)	130.87 (7.29)	0.00
DBP (Mean, SD)	78.74 (11.53)	78.10 (10.33)	0.06	78.52 (9.82)	78.52 (4.55)	0.00
Fasting blood glucose (Mean, SD)	115.61 (49.21)	103.74 (29.28)	0.29	110.20 (33.15)	110.20 (18.33)	0.00
Total cholesterol (Mean, SD)	190.26 (45.68)	193.11 (38.84)	0.07	190.61 (39.05)	190.61 (16.87)	0.00
CCI score (Mean, SD)	2.19 (2.20)	1.13 (1.73)	0.53	1.84 (1.68)	1.84 (0.99)	0.00
SSNHL (*n*, %)	153 (0.92)	603 (0.90)	0.00	110 (0.89)	109 (0.88)	0.00

Abbreviations: CCI, Charlson Comorbidity Index; CKD, chronic kidney disease; SBP, systolic blood pressure; DBP, diastolic blood pressure; SSNHL, sudden sensorineural hearing loss. † Obesity (BMI, body mass index, kg/m^2^) was categorized as <18.5 (underweight), ≥18.5 to <23 (normal), ≥23 to <25 (overweight), ≥25 to <30 (obese I), and ≥30 (obese II).

**Table 2:** Crude and overlap propensity score weighted hazard ratios (95% confidence interval) of CKD for SSNHL with subgroup analyses according to age, sex, income, region of residence, obesity, smoking, alcohol consumption, SBP, DBP, fasting blood glucose, total cholesterol, and CCI scores.

	N of Event/ N of Total (%)	Follow-Up Duration (PY)	IR per 1000 (PY)	IRD (95% CI)	Hazard Ratios for SSNHL			
					Crude	*p*-Value	Overlap Weighted Model †	*p*-Value
Total participants								
CKD	153/16,713 (0.92)	70,780	2.16	0.42 (0.07 to 0.76)	1.22 (1.02 to 1.45)	0.031 *	1.21 (1.04 to 1.39)	0.011 *
Control	603/66,852 (0.90)	346,022	1.74		1		1	
Age < 70 years old								
CKD	118/8,083 (1.46)	45,337	2.60	0.67 (0.21 to 1.13)	1.33 (1.09 to 1.63)	0.006 *	1.30 (1.10 to 1.54)	0.002 *
Control	414/32,332 (1.28)	214,572	1.93		1		1	
Age ≥ 70 years old								
CKD	35/8630 (0.41)	25,443	1.38	−0.06 (−0.57 to 0.45)	0.90 (0.63 to 1.30)	0.580	0.95 (0.72 to 1.25)	0.724
Control	189/34,520 (0.55)	131,450	1.44		1		1	
Male								
CKD	98/10,981 (0.89)	45,558	2.15	0.31 (−0.12 to 0.75)	1.15 (0.92 to 1.44)	0.212	1.18 (0.99 to 1.41)	0.070
Control	408/43,924 (0.93)	222,167	1.84		1		1	
Female								
CKD	55/5732 (0.96)	25,222	2.18	0.61 (0.05 to 1.16)	1.35 (1.00 to 1.82)	0.048 *	1.26 (0.98 to 1.62)	0.069
Control	195/22,928 (0.85)	123,855	1.57		1		1	
Low-income group								
CKD	60/7236 (0.83)	30,267	1.98	0.25 (−0.26 to 0.78)	1.12 (0.85 to 1.49)	0.421	1.07 (0.85 to 1.34)	0.579
Control	260/28,944 (0.90)	150,599	1.73		1		1	
High-income group								
CKD	93/9477 (0.98)	40,513	2.30	0.54 (0.08 to 1.00)	1.28 (1.02 to 1.61)	0.033 *	1.31 (1.09 to 1.58)	0.005 *
Control	343/37,908 (0.90)	195,423	1.76		1		1	
Urban resident								
CKD	83/7157 (1.16)	32,171	2.58	0.77 (0.24 to 1.30)	1.41 (1.10 to 1.80)	0.006 *	1.39 (1.13 to 1.70)	0.002 *
Control	279/28,628 (0.97)	154,461	1.81		1		1	
Rural resident								
CKD	70/9556 (0.73)	38,609	1.81	0.12 (−0.33 to 0.57)	1.05 (0.81 to 1.36)	0.728	1.05 (0.85 to 1.29)	0.647
Control	324/38,224 (0.85)	191,561	1.69		1		1	
Underweight								
CKD	2/443 (0.45)	1359	1.47	0.11 (−2.00 to 2.22)	1.06 (0.24 to 4.70)	0.940	1.05 (0.34 to 3.19)	0.937
Control	13/2220 (0.59)	9526	1.36		1		1	
Normal weight								
CKD	47/5129 (0.92)	20,969	2.24	0.65 (0.05 to 1.26)	1.38 (1.00 to 1.89)	0.049 *	1.29 (1.00 to 1.67)	0.049 *
Control	195/23,822 (0.82)	122,872	1.59		1		1	
Overweight								
CKD	52/4379 (1.19)	19,568	2.66	0.74 (0.04 to 1.43)	1.36 (1.00 to 1.85)	0.052	1.30 (1.01 to 1.68)	0.043 *
Control	183/18,037 (1.01)	95,092	1.92		1		1	
Obese								
CKD	52/6762 (0.77)	28,884	1.80	0.01 (−0.53 to 0.56)	0.99 (0.73 to 1.34)	0.948	1.05 (0.82 to 1.35)	0.686
Control	212/22,773 (0.93)	118,532	1.79		1		1	
Nonsmoker								
CKD	112/10,688 (1.05)	46,836	2.39	0.63 (0.20 to 1.06)	1.33 (1.08 to 1.65)	0.007 *	1.29 (1.09 to 1.54)	0.004 *
Control	399/43,275 (0.92)	226,976	1.76		1		1	
Past and current smoker								
CKD	41/6025 (0.68)	23,944	1.71	0.00 (−0.58 to 0.57)	0.98 (0.70 to 1.37)	0.888	1.00 (0.77 to 1.29)	0.990
Control	204/23,577 (0.87)	119,046	1.71		1		1	
Alcohol consumption < 1 time a week								
CKD	116/12,152 (0.95)	52,397	2.21	0.44 (0.04 to 0.85)	1.22 (1.00 to 1.50)	0.053	1.20 (1.01 to 1.42)	0.037 *
Control	428/46,344 (0.92)	241,865	1.77		1		1	
Alcohol consumption ≥ 1 time a week								
CKD	37/4561 (0.81)	18,383	2.01	0.33 (−0.32 to 0.98)	1.18 (0.83 to 1.68)	0.360	1.21 (0.92 to 1.59)	0.167
Control	175/20,508 (0.85)	104,157	1.68		1		1	
SBP < 140 mmHg and DBP < 90 mmHg								
CKD	102/10,953 (0.93)	44,649	2.28	0.45 (0.01 to 0.89)	1.22 (0.98 to 1.51)	0.073	1.23 (1.04 to 1.46)	0.015 *
Control	451/48,853 (0.92)	246,199	1.83		1		1	
SBP ≥ 140 mmHg or DBP ≥ 90 mmHg								
CKD	51/5760 (0.89)	26,131	1.95	0.43 (−0.12 to 0.98)	1.27 (0.92 to 1.74)	0.145	1.11 (0.84 to 1.47)	0.460
Control	152/17,999 (0.84)	99,823	1.52		1		1	
Fasting blood glucose < 100 mg/dL								
CKD	69/7801 (0.88)	35,843	1.93	0.19 (−0.29 to 0.65)	1.08 (0.84 to 1.40)	0.552	1.10 (0.91 to 1.34)	0.313
Control	363/37,239 (0.97)	208,139	1.74		1		1	
Fasting blood glucose ≥ 100 mg/dL								
CKD	84/8912 (0.94)	34,937	2.40	0.66 (0.16 to 1.17)	1.36 (1.06 to 1.74)	0.016 *	1.31 (1.05 to 1.63)	0.015 *
Control	240/29,613 (0.81)	137,883	1.74		1		1	
Total cholesterol < 200 mg/dL								
CKD	86/10,354 (0.83)	40,676	2.11	0.41 (−0.04 to 0.86)	1.22 (0.96 to 1.55)	0.100	1.22 (1.01 to 1.49)	0.040 *
Control	330/39,341 (0.84)	193,809	1.70		1		1	
Total cholesterol ≥ 200 mg/dL								
CKD	67/6359 (1.05)	30,104	2.23	0.44 (−0.10 to 0.97)	1.22 (0.93 to 1.59)	0.151	1.20 (0.96 to 1.49)	0.104
Control	273/27,511 (0.99)	152,213	1.79		1		1	
CCI scores = 0								
CKD	52/4877 (1.07)	22,865	2.27	0.45 (−0.14 to 1.05)	1.23 (0.92 to 1.64)	0.165	1.23 (1.01 to 1.49)	0.039 *
Control	348/35,944 (0.97)	191,409	1.82		1		1	
CCI scores = 1								
CKD	23/2844 (0.81)	11,499	2.00	0.12 (−0.75 to 0.98)	1.04 (0.66 to 1.62)	0.872	0.98 (0.70 to 1.37)	0.918
Control	120/12,306 (0.98)	63,790	1.88		1		1	
CCI scores ≥ 2								
CKD	78/8992 (0.87)	36,416	2.14	0.65 (0.16 to 1.15)	1.42 (1.08 to 1.88)	0.013 *	1.24 (0.94 to 1.64)	0.129
Control	135/18,602 (0.73)	90,823	1.49		1		1	

Abbreviation: CCI, Charlson Comorbidity Index; SBP, systolic blood pressure; DBP, diastolic blood pressure; CKD, chronic kidney disease; SSNHL, sudden sensorineural hearing loss; IR, incidence rate; IRD, incidence rate difference; PY person-year; CI, confidence interval. * Significance at *p* < 0.05 † adjusted for age, sex, income, region of residence, obesity, smoking, alcohol consumption, SBP, DBP, fasting blood glucose, total cholesterol, and CCI scores.

## Data Availability

Restrictions apply to the availability of these data. Data was obtained from Health Insurance Review and Assessment Service (HIRA) of Korea and are available at https://opendata.hira.or.kr with the permission of HIRA.
